# Simultaneous inhibition of FAK and ROS1 synergistically repressed triple-negative breast cancer by upregulating p53 signalling

**DOI:** 10.1186/s40364-024-00558-0

**Published:** 2024-01-25

**Authors:** Ximin Tan, Deguang Kong, Zhuoli Tao, Fangling Cheng, Baoyuan Zhang, Zaiqi Wang, Qi Mei, Chuang Chen, Kongming Wu

**Affiliations:** 1 Department of Oncology, Tongji Hospital of Tongji Medical College, Huazhong University of Science and Technology, Wuhan, 430030 China; 2 Department of Breast and Thyroid Surgery, Renmin Hospital of Wuhan University, 238 Ziyang Road, Wuhan, 430060 China; 3 Department of Breast and Thyroid Surgery, Tongji Hospital of Tongji Medical College, Huazhong University of Science and Technology, Wuhan, 430030 China; 4 Hepatic Surgery Center, Tongji Hospital of Tongji Medical College, Huazhong University of Science and Technology, Wuhan, 430030 China; 5 InxMed (Shanghai) Co. Ltd, Shanghai, China; 6 Cancer Center, Shanxi Bethune Hospital, Shanxi Academy of Medical Science, Tongji Shanxi Hospital, Third Hospital of Shanxi Medical University, Taiyuan, 030032 China; 7 Cancer Center, Tongji Hospital of Tongji Medical College, Huazhong University of Science and Technology, Wuhan, 430030 China

**Keywords:** FAK, ROS1, TNBC, Ferroptosis, Combination therapy, Organoid

## Abstract

**Background:**

Triple-negative breast cancer (TNBC) is an aggressive breast cancer subtype lacking effective targeted therapies, necessitating innovative treatment approaches. While targeting ROS proto-oncogene 1 (ROS1) with crizotinib has shown promise, resistance remains a limitation. Recent evidence links focal adhesion kinase (FAK) to drug resistance, prompting our study to assess the combined impact of FAK inhibitor IN10018 and crizotinib in TNBC and elucidate the underlying mechanisms.

**Methods:**

We employed the Timer database to analyze FAK and ROS1 mRNA levels in TNBC and adjacent normal tissues. Furthermore, we investigated the correlation between FAK, ROS1, and TNBC clinical prognosis using the GSE database. We conducted various in vitro assays, including cell viability, colony formation, flow cytometry, EdU assays, and western blotting. Additionally, TNBC xenograft and human TNBC organoid models were established to assess the combined therapy’s efficacy. To comprehensively understand the synergistic anti-tumor mechanisms, we utilized multiple techniques, such as RNA sequencing, immunofluorescence, cell flow cytometry, C11-BODIPY staining, MDA assay, and GSH assay.

**Results:**

The Timer database revealed higher levels of FAK and ROS1 in TNBC tissues compared to normal tissues. Analysis of GEO databases indicated that patients with high FAK and ROS1 expression had the poorest prognosis. Western blotting confirmed increased p-FAK expression in crizotinib-resistant TNBC cells. In vitro experiments showed that the combination therapy down-regulated cyclin B1, p-Cdc2, and Bcl2 while up-regulating BAX, cleaved-Caspase-3, cleaved-Caspase-9, and cleaved PARP. In TNBC xenograft models, the tumor volume in the combination therapy group was 73% smaller compared to the control group (*p* < 0.0001). Additionally, the combination therapy resulted in a 70% reduction in cell viability in human TNBC organoid models (*p* < 0.0001). RNA sequencing analysis of TNBC cells and xenograft tumor tissues highlighted enrichment in oxidative stress, glutathione metabolism, and p53 pathways. The combined group displayed a fivefold rise in the reactive oxygen species level, a 69% decrease in the GSH/GSSG ratio, and a sixfold increase in the lipid peroxidation in comparison to the control group. Western blotting demonstrated p53 upregulation and SCL7A11 and GPX4 downregulation in the combination group. The addition of a p53 inhibitor reversed these effects.

**Conclusion:**

Our study demonstrates that the combination of IN10018 and crizotinib shows synergistic antitumor effects in TNBC. Mechanistically, this combination inhibits cell proliferation, enhances apoptosis, and induces ferroptosis, which is associated with increased p53 levels.

## Introduction

Breast cancer ranks as the leading malignancy among women and stands as the second most prevalent reason for cancer-related deaths globally [1, 2]. This complex disease comprises various subtypes, including hormone receptor-positive breast cancer, human epidermal growth factor receptor 2 (HER2)-positive breast cancer, and the notably aggressive triple-negative breast cancer (TNBC) [3, 4]. Traditional therapies targeting endocrine or HER2 receptors prove ineffective in TNBC due to the absence of relevant receptor markers [5]. Presently, chemotherapy remains the primary treatment for TNBC, but 60–70% of patients develop drug resistance, leading to tumor recurrence and metastasis [6, 7]. Consequently, there’s a pressing need to explore new strategies for TNBC [8–11].

Notably, approximately 73% of TNBC patients exhibit E-cadherin deficiency [12]. Ilirjana Bajrami and colleagues’ study unveiled a remarkable discovery, revealing synthetic lethality between the inhibition of ROS proto-oncogene 1 (ROS1) and the E-cadherin-deficient breast cancer subtype. Crizotinib, in particular, yielded encouraging results in this context [13]. This highlights the potential of ROS1 inhibition as a profound and effective therapeutic strategy for TNBC.

ROS1, situated on chromosome 6 and belonging to the insulin receptor family, functions as a receptor tyrosine kinase [14]. The primary pathogenic mutation in the ROS1 gene involves gene rearrangements, resulting in the formation of fusion proteins [15]. Various fusion partners of ROS1 kinase fusions have been identified in different cancers, with CD74 being the most prevalent [16]. These fusions often lead to the continuous activation of the ROS1 tyrosine kinase region and downstream signaling pathways, including MAPK/ERK, PI3K/AKT/mTOR, and JAK/STAT, promoting cell proliferation and tumorigenesis [17]. Initially observed in glioblastoma multiforme, ROS1 rearrangements have since been identified in numerous cancer types, including cholangiocarcinoma, gastric cancer, ovarian cancer [18], and breast cancer [19]. The use of ROS1 inhibitors in ROS1-positive patients has yielded promising results, extending survival in cases like atypical meningioma [20] and lung blastoma [21]. Given the favorable clinical activity of crizotinib, ROS1 TKI therapy has become the first-line treatment for advanced non-small cell lung cancer (NSCLC) patients initially testing positive for ROS1 fusion [22, 23]. Furthermore, combining ROS1-targeted therapies with other treatments, such as crizotinib and radiotherapy for brain-metastatic lung adenocarcinoma, holds promise for improving patient outcomes [24]. In summary, ROS1-targeted therapies have shown substantial clinical efficacy, both as monotherapies and in combination with other treatments, underscoring their potential as a therapeutic approach for patients with various tumor types.

Focal adhesion kinase (FAK), a cytoplasmic non-receptor tyrosine kinase, is essential in transmitting signals from the extracellular matrix (ECM) to the cell cytoplasm. Integrin binding to the ECM results in autophosphorylation at the Tyr397 site, crucial for the cellular functions of FAK [25]. A wealth of evidence underscores the association between FAK and cancer [26, 27], leading to the development of numerous FAK inhibitors with efficacy in suppressing tumor growth and metastasis [28–30]. Current research focuses on the connection between FAK and resistance to treatment in various cancer types. In KRAS-mutated cancers, the combination of FAK inhibitors and KRAS G12C inhibitors exhibits promise in reducing drug resistance [31]. In NSCLC, FAK inhibition effectively delays the development of osimertinib-resistant tumors [32]. Additionally, the combined use of platinum and FAK inhibitors shows potential in treating chemotherapy-resistant ovarian cancers [33]. It’s worth noting that in TNBC, inhibiting FAK/Src makes cells more susceptible to chemotherapy [34]. IN10018 is a novel and highly selective FAK-Y397 inhibitor, as known as BI 853520. Studies have shown its efficacy in various cancers such as breast cancer and oesophageal cancer [35–37]. IN10018 has been granted fast track designation by the U.S. Food and Drug Administration and has also received Breakthrough Therapy Designation from the China National Medical Products Administration.

Given the increasing evidence linking FAK to drug resistance in cancer, we explored the feasibility of combining IN10018 with crizotinib for the treatment of TNBC.

## Materials and methods

### Cell lines

The cells were acquired from the Oncology Laboratory, Tongji Hospital, Huazhong University of Science and Technology. Human breast cancer cells MDA-MB-231 and Hs578T were cultured in DMEM (Hyclone, USA) supplemented with 10% fetal bovine serum (Gibco, USA) and 1% penicillin–streptomycin (Gibco, USA). Ba/F3-WT cells were cultured in RMPI-1640 (Hyclone, USA) supplemented with 10% fetal bovine serum (Gibco, USA), 1% penicillin–streptomycin (Gibco, USA), and IL-3 (1 ng/mL). Ba/F3-CD74-ROS1 cells were cultured in RMPI-1640 (Hyclone, USA) supplemented with 10% fetal bovine serum (Gibco, USA) and 1% penicillin–streptomycin. Ba/F3-WT cells and Ba/F3-CD74-ROS1 cells were provided by Precedo (Anhui, China). All of the cells were cultured in a humidified incubator at 37 °C with 5% CO_2_.

### Reagents

IN10018, a FAK inhibitor, was provided by InxMed (Shanghai). The ROS1 inhibitor Crizotinib, the ferroptosis inhibitor Ferrostain-1(Fer-1), and the p53 inhibitor Pifithrin-α were acquired from MedChemExpress.

### Cell viability assay

Cells of the logarithmic growth phase (10,000 cells per well) were seeded in 24-well plates. After 48 h of drug treatment, the control cells grew near to full confluence, the cells were digested for cell counting.

### Colony formation assay

In the colony formation assay, cells were plated in six-well plates (1000 cells/well). Three replicate wells were set up for each group and placed in an incubator overnight. The medium was added with different concentrations of the drugs. After 10–14 days of incubation, colonies were immobilized utilizing 4% paraformaldehyde fixation, then adding 0.2% (w/v) crystal violet stain. Quantification of colonies was accomplished utilizing the Image J software.

### Cell apoptosis analysis by flow cytometry

The apoptosis efficacy was assessed by an Annexin V-FITC/PI Apoptosis Detection Kit (Yeasen, China). After different treatments, 5 × 10^5^ cells were obtained. Cells were stained with FITC Annexin V and PI staining solution, followed by a 15-min incubation at room temperature. Before detection, the sample was mixed with 400 μL of 1 × binding buffer and placed on ice. Flow cytometry was performed within 1 h, and the results were analyzed using FlowJo V10 software.

### Western blotting

Washing the cells twice with PBS, they were lysed with RIPA buffer on ice for 30 min. After centrifugation, the cell lysates were loaded onto a 10% SDS–polyacrylamide gel and subsequently transferred to PVDF membranes. For further analysis, the membranes were incubated with the primary antibodies: p-ROS1 (TA8186, Abmart), ROS1 (TD10344, Abmart), p-FAK (8556, Cell Signaling Technology), FAK (66,258–1-Ig, ProteinTech), p53 (2527, Cell Signaling Technology), cyclin B1 (595, Santa Cruz Biotechnology), p-Cdc2 (12,340, Santa Cruz Biotechnology), Cdc2 (A0220, Abclonal), Bcl-2 (T40056, Abmart), Survivin (2808, Cell Signaling Technology), BAX (T40051, Abmart), cleaved PARP (9541, Cell Signaling Technology), PARP (9542, Cell Signaling Technology), cleaved-Caspase-9 (9509, Cell Signaling Technology), Caspase 9 (9508, Cell Signaling Technology), cleaved-Caspase-3 (9664, Cell Signaling Technology), Caspase 3 (9662, Cell Signaling Technology), SLC7A11 (12,691, Cell Signaling Technology), GPX4 (59,735, Cell Signaling Technology), p21 (397, Santa Cruz Biotechnologies Biotechnology), p27 (25,614–1-AP, ProteinTech), GAPDH (60,004–1-Ig, ProteinTech) overnight at 4 °C. The cell membranes were incubated with Affinipure Goat Anti-Mouse IgG (SA00001-1, ProteinTech) or Affinipure Goat Anti-Rabbit IgG (SA00001-2, ProteinTech) secondary antibodies conjugated with horseradish peroxidase (HRP) for 1 h at room temperature. The membranes were then exposed to the Syngene G: BOX Chemi XT4 imaging system (Britain).

### Bioinformatics analysis

The TIMER database (https://cistrome.shinyapps.io/timer/) is an extensive resource for analyzing mRNA levels in tumors and paracancers. The GSE159956 and GSE10885 datasets were obtained from the Gene Expression Omnibus (GEO) (http://www.ncbi.nlm.nih.gov/geo) to analyze the prognostic implications of FAK and ROS1 in breast cancer.

### Analysis of combined drug effects

The combination index (CI) of drugs was calculated using CompuSyn software. A CI value below 1 indicates a synergistic effect, while a CI value equal to 1 or greater than 1 indicates additive and antagonist effects, respectively.

### 5-ethynyl-2′-deoxyuridine assay (EdU)

EdU assay was performed using the Cell-Light EdU Apollo 567 In Vitro Imaging Kit (RiboBio, Guangzhou, China). Cells were plated in 24-well plates at 2 × 10^6^ cells/well. After 24 h, the cells were given different drugs based on the experimental design. To examine cell growth, the cell culture medium was enriched with 50 µM of EdU and subsequently subjected to an incubation period at 37˚C for 2 h. The cells were washed with PBS twice and then fixed with 4% absolute methanol for 30 min at room temperature. Following fixation, cells were incubated with 2 mg/ml glycine for 5 min followed by washing with phosphate-buffered solution (PBS). After permeabilizing the cells with PBS containing 0.5% Triton X-100 and performing a subsequent wash with PBS, the cells were exposed to Apollo staining solution for 30 min at room temperature in a light-proof shaker. Next, the cells were washed 2 times with PBS containing 0.5% Triton X-100. Lastly, the cells were subjected to Hoechst 33,342 staining for 30 min at room temperature, followed by three PBS washes before imaging under a fluorescent microscope.

### Cell cycle analysis by flow cytometry

The cell cycle efficacy was evaluated using the Cell Cycle and Apoptosis Analysis Kit (Yeasen, China). After different treatments, 1 × 10^5^ ~ 1 × 10^6^ cells were obtained, washed with PBS, and fixed by prechilled 70% ethanol for 2 h at 4 °C. After washing cells with PBS, the pellet was resuspended in 0.5 ml 1 × binding buffer containing 10 μl PI and 10 μl RNase A. Next, cells were kept in the dark at room temperature for 30 min. Samples were analyzed using flow cytometry, and the results were analyzed by using the Modfit software.

### Mouse xenograft models

Female BALB/c mice were subcutaneously inoculated with 3 × 10^6^ MDA-MB-231 cells. When tumors reached 50–100 mm^3^, animals were randomized into four groups: control, IN10018, crizotinib, and combination group. The control group received 0.5% natrosol 250 HX treatment by intragastric administration once a day. The IN10018 or crizotinib group was given intragastric administration of 25 mg/kg once a day. All animal experiments were approved by the Ethics Committee of Tongji Hospital, Huazhong University of Science and Technology (TJH-202006010).

### Immunohistochemistry

Immunohistochemistry was conducted on formalin-fixed and paraffin-embedded mouse tumor tissue as previously described [38]. After de-paraffinization and rehydration, sections were placed in boiling citric acid repair solution for 5 min and then heated in a microwave oven at low temperature for 20 min. The sections were incubated in a wet box of 3% H_2_O_2_, washed 3 times with PBS, and then closed with 10% sheep serum at room temperature for 30 min. The corresponding primary antibody was added and incubated overnight at 4 °C in a wet box. Then the antibody was incubated with a biotin-labeled secondary antibody for 30 min. Then DAB dye was added dropwise and the color development was observed under a microscope. The staining was terminated by placing it in water when the color was moderate. The stain was then re-stained with hematoxylin. The pathologist scoring the immunohistochemistry was blinded to the data, with five high-magnification fields per slide and 100 cells counted in each field for analysis.

### RNA-seq

Novogene (Beijing, China) performed the following RNA-seq analyses. Genes with adjusted *P*-value < 0.05 and absolute log2 fold changes > 0 were identified as differentially expressed. First, total RNA was isolated from MDA-MB-231 cells and MDA-MB-231 tumor tissues using Trizol (Takara Bio). Subsequently, RNA was purified, and library preparation and sequencing were performed on the Illumina Hiseq platform. The resultant data from the three datasets were analyzed for differential expression using the DESeq2 R software package (version 1.20.0). Genes were considered to be significantly differentially expressed if their p-values were less than 0.05. Further analyses of these differentially expressed genes (DEGs) included gene ontology (GO) and Kyoto Encyclopedia of the Genome (KEGG) pathway analyses, which were performed using the clusterProfiler R software package.

### Tumor cell isolation and 3D organoid cultures

Breast cancer specimens were obtained from the Renmin Hospital of Wuhan University. The study was approved by the ethics committee of Renmin Hospital, Wuhan University (approved number: WDRY2022-K002). The surface mucus and necrotic tissue of the tumor sample were removed before washing with PBS, and the tissue was soaked with active iodine for 1 min. Subsequently, samples were rinsed thrice with penicillin/streptomycin. Then, the tissue was chopped into 0.5-mm pieces, digested in 50% TrypLE™ Express (12,605,028, Gibco)/50% DMEM at 37 °C for 15 min, and neutralized with the same volume of Ca^2+^ containing Hanks’ Balanced Salt solution. The cell suspension was allowed to settle for 3–5 min, and the supernatant was collected, and centrifuged. The pellet was washed twice in advanced DMEM/F12, counted, and mixed with growth factor-reduced Matrigel. In a pre-warmed 24-well plate, 8,000–10,000 cells were seeded per well with 30 µl Matrigel. After Matrigel solidification following incubation at 37 °C for 30 min, 600 µl of breast cancer organoid medium was overlaid, and then the plate into a 37 °C and 5% CO_2_ incubator.

### Organoids proliferation

When the breast cancer organoids entered the logarithmic phase, they were subjected to mechanical and enzymatic dissociation by incubating them in TrypLE (12,604,013, Gibco) for 10–14 min, and then resuspended in Matrigel at a concentration of 3 × 10^5^/ml separately on ice. To conduct the proliferation analysis, the suspensions were inoculated onto pre-warmed 96-well plates with 3 × 10^3^ cells per well and maintained in a complete culture medium (100 µl/well). Cell viability was quantified using 50 μl of CellTiter-Glo 3D (G9681, Promega) mixed with 50 μl of culture medium, following the manufacturer's instructions. Medium was replaced with this mixture, and the quantification was performed on an Infinite 200 Pro plate reader (Tecan Life Sciences). GraphPad Prism 8 was used to analyze organoid proliferation rates and generate graphs.

### Assessment of reactive oxygen species (ROS)

Intracellular levels of ROS were quantified using 2′,7′-dichlorofluorescein diacetate (DCFH-DA). Following 24 h of treatment with or without drugs, the cells underwent three washes with serum-free medium. Subsequently, a 10 μM concentration of DCFH-DA dye (Beyotime, S0033S) was added to each well and incubated at 37 °C for 20 min. After that, cells were subjected to three washes with serum-free medium and then either captured pictures or collected for flow cytometry. All experiments were conducted in triplicate.

### Lipid peroxidation assay

Lipid peroxidation reactions were identified by the BODIPY™ 581/591 C11 (Thermo Fisher Scientific). Notably, unoxidized cells bear a bright red fluorescent marker, while oxidized cells show a distinct green fluorescent marker. Lipid peroxidation levels in cellular systems were assessed and characterized by fluorescent labeling.

### Glutathione (GSH) quantification

To determine the GSH content, we utilized the GSH Assay Kit (#A006-2–1) sourced from the Nanjing Institute of Architectural Bioengineering in China. Absorbance values were measured at 405 nm. The calculated quantities of reduced GSH, oxidized glutathione (GSSG), and total glutathione (T-GSH) were derived using the specified formula.

### Malondialdehyde (MDA) assay

The MDA Assay Kit (Solarbio, Beijing, China) was utilized to determine the quantity of MDA in the cells. When MDA interacts with thiobarbituric acid, it produces a brown–red complex, and the intracellular content of MDA may be measured by quantifying the cells' absorbance at 532 and 600 nm.

### Statistical analysis

This study was statistically analyzed using GraphPad Prism 8. ANOVA was used to compare the four groups, and the Student's t-test was used to compare the two groups. All statistical analysis was performed with statistical significance determined as follows: ns = not significant (*P* > 0.05), * *P* < 0.05, ** *P* < 0.01, and *** *P* < 0.001.

## Results

### p-FAK was upregulated in crizotinib-resistant TNBC cells

**Fig. 1:**
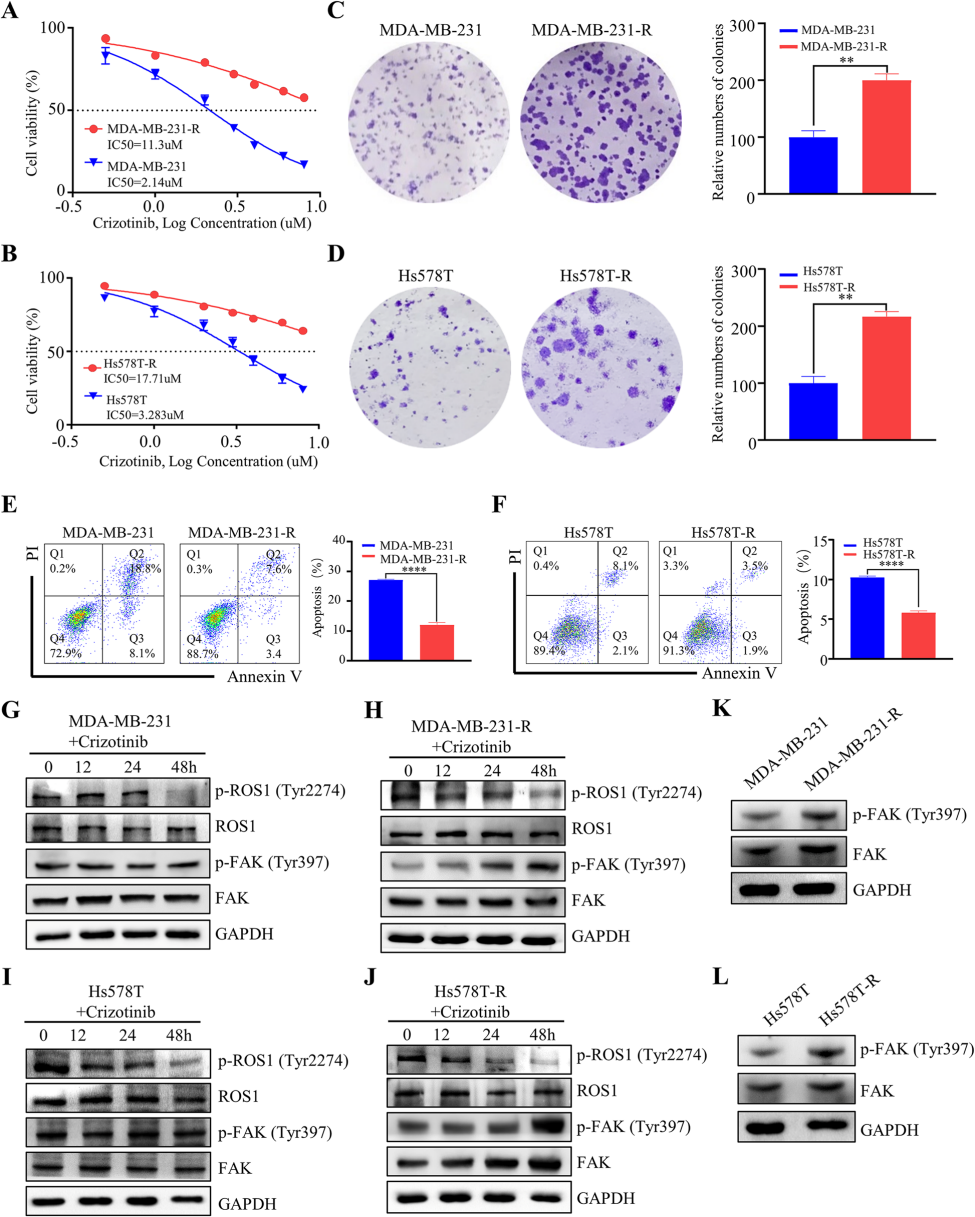
p-FAK was upregulated in crizotinib-resistant TNBC cells. **A** The cell viability and IC50 of MDA-MB-231 parental cell line and crizotinib-resistant line (MDA-MB-231-R) following a 48-h incubation with various concentrations of crizotinib. **B** The cell viability and IC50 of the Hs578T parental cell line and crizotinib-resistant line (Hs578T-R) following a 48-h incubation with various concentrations of crizotinib. **C**, **D** Representative figures (left) and quantitative analysis (right) of colony formation assay of MDA-MB-231 and Hs578T parental cells, as well as crizotinib-resistant cells. **E**, **F** Representative figures (left) and quantitative analysis (right) of apoptosis measured by flow cytometry of MDA-MB-231 and Hs578T parental cells, as well as crizotinib-resistant cells. **G**, **H**, **I**, **J** Western blotting results of MDA-MB-231 and Hs578T parental cells, as well as crizotinib-resistant cells. **K**, **L** The p-FAK levels between parental and crizotinib-resistant cells. Data are represented as mean ± SEM. Significant levels: ns = not significant (*P* > 0.05), * *P* < 0.05, ** *P* < 0.01, and *** *P* < 0.001

To investigate the p-FAK levels of crizotinib resistance in TNBC cells, we established crizotinib-resistant cells by gradually increasing the concentration of crizotinib for 4 months. The IC50 value for the MDA-MB-231 parental cells was determined to be 2.14 μM, whereas the crizotinib-resistant cells displayed a significantly higher IC50 value of 11.3 μM (Fig. 1A). Similarly, Hs578T parental cells exhibited an IC50 value of 3.283 μM, while the crizotinib-resistant cells showed an elevated IC50 value of 17.71 μM (Fig. 1B). To compare the growth rates of parental and crizotinib-resistant cells, the cells were exposed to the IC50 concentration of crizotinib in the parental cells. The results of colony formation demonstrated that crizotinib-resistant cells exhibited faster cell proliferation in MDA-MB-231 (Fig. 1C) and Hs578T cells (Fig. 1D) compared to parental cells. Additionally, the apoptosis results indicated that crizotinib-resistant cells displayed greater resistance to apoptosis (Fig. 1E, F). To investigate the correlation between FAK and crizotinib resistance, we analyzed the levels of p-FAK in MDA-MB-231 (Fig. 1G, H) and Hs578T cells (Fig. 1I, J) at various times (0, 12, 24, and 48 hours) after incubation with IC50 of crizotinib. Our findings revealed a sequential decrease in the levels of p-ROS1 in both MDA-MB-231 and Hs578T cells as the incubation time increased. Conversely, p-FAK levels remained relatively unchanged in parental cells, while they increased over time in the crizotinib-resistant cells. Crizotinib-resistant cells showed higher levels of p-FAK than parental cells. (Fig. 1K, L). Our research indicated that the resistance to crizotinib in TNBC was linked to an increase in p-FAK, which in turn leads to enhanced cell proliferation and decreased apoptosis.

### The co-expression of FAK and ROS1 was associated with the poorest prognosis, whereas the simultaneous inhibition of FAK and ROS1 suppressed cell growth synergistically

**Fig. 2:**
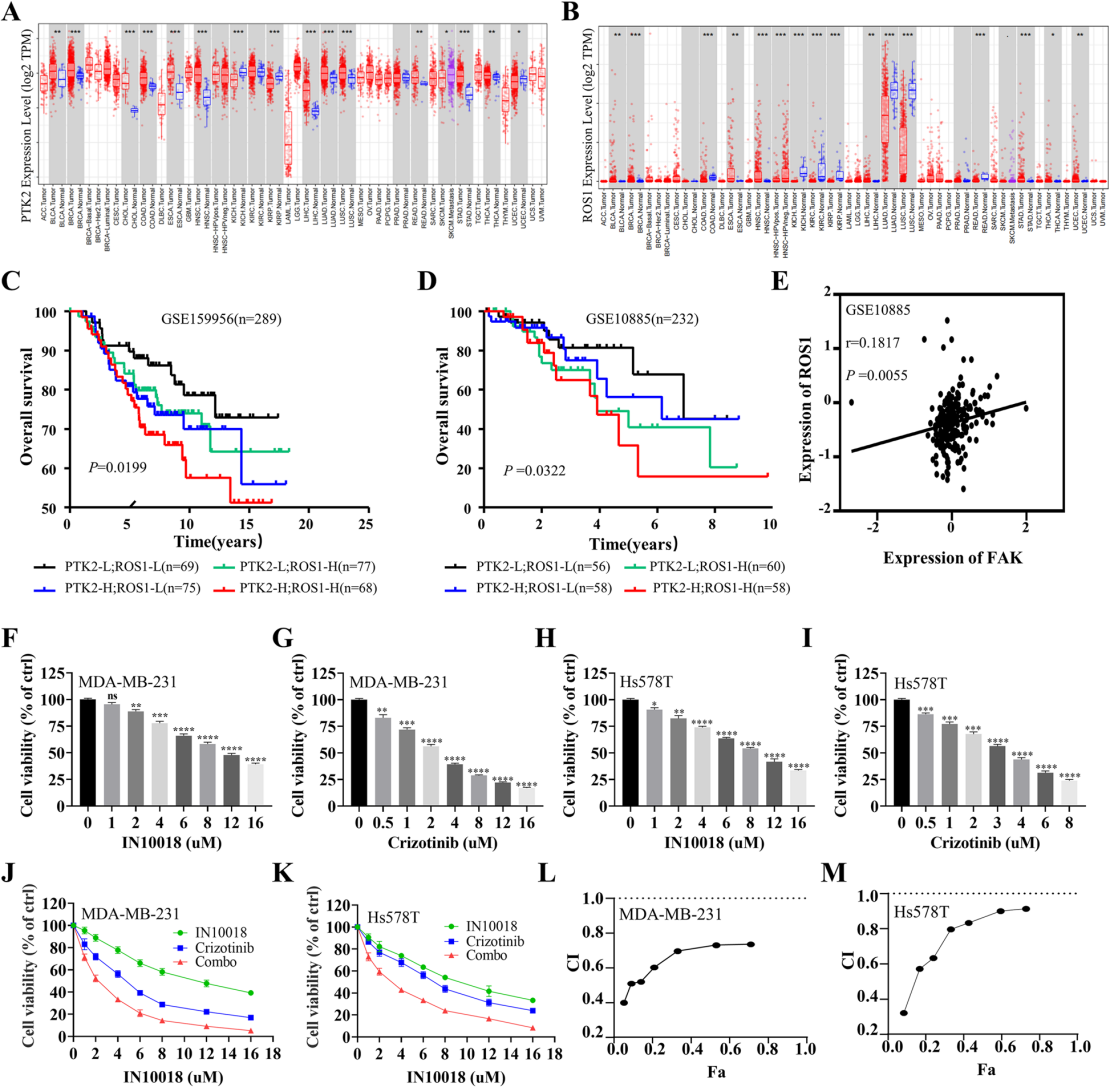
The co-expression of FAK and ROS1 was associated with the poorest prognosis, whereas the simultaneous inhibition of FAK and ROS1 suppressed cell growth synergistically. **A**, **B** The mRNA expression levels of FAK (PTK2) and ROS1 in breast cancer tissues and normal breast tissues were analyzed by the TIMER database. **C**, **D** Survival curves of OS in breast cancer patients from GSE159956 and GSE10885. **E** Correlation analysis of FAK and ROS1. **F**, **G** The cell viability of MDA-MB-231 cells treated with serial doses of IN10018 and crizotinib. **H**, **I** The cell viability of Hs578T cells treated with serial doses of IN10018 and crizotinib. **J**, **K** Survival curves of MDA-MB-231 and Hs578T cells treated with IN10018, crizotinib, or combination therapy. **L**, **M** The CI value of MDA-MB-231 and Hs578T cells treated with IN10018, crizotinib, or combination therapy. Data are represented as mean ± SEM. Significant levels: ns = not significant (*P* > 0.05), * *P* < 0.05, ** *P* < 0.01, and *** *P* < 0.001. L: Low, H: High

To investigate the clinical significance between FAK and ROS1 in TNBC, we conducted a comprehensive survey of mRNA levels in breast cancer and normal breast tissue. According to the TIMER database, the levels of FAK (Fig. 2A) and ROS1 (Fig. 2B) were higher in breast cancer tissue in comparison to normal breast tissue. To further understand the clinical implications of FAK and ROS1 expression, we performed an in-depth investigation of patient prognosis using the GSE159956 (Fig. 2C) and GSE10885 (Fig. 2D) datasets. Database suggested that FAK and ROS1 are positively correlated (Fig. 2E). The results revealed a correlation between elevated levels of FAK or ROS1 expression and reduced OS in breast cancer patients. Interestingly, patients with elevated levels of both FAK and ROS1 had the worst prognosis compared to those with elevated expression of either FAK or ROS1 alone. The cell viability assays were conducted to determine the inhibitory impact of IN10018 and crizotinib on MDA-MB-231 (Fig. 2F, G) and Hs578T cells (Fig. 2H, I). The results demonstrated that the inhibitory effect on TNBC cell growth increased as the concentration increased with IN10018 or crizotinib. Notably, a higher level of inhibition was observed when both drugs were used together compared to using each drug alone (Fig. 2J, K). To further investigate this effect, the CI values of the drugs were calculated. Interestingly, TNBC cells in this study had CI values less than 1 (Fig. 2L, M), strongly supporting the synergistic anticancer effects of IN10018 and crizotinib on TNBC cells. These findings provide valuable insights into the combination of IN10018 and crizotinib for the treatment of TNBC.

### Crizotinib exhibited greater sensitivity towards Ba/F3-CD74-ROS1 cells compared to Ba/F3-WT cells, and its efficacy was enhanced when combined with IN10018

**Fig. 3:**
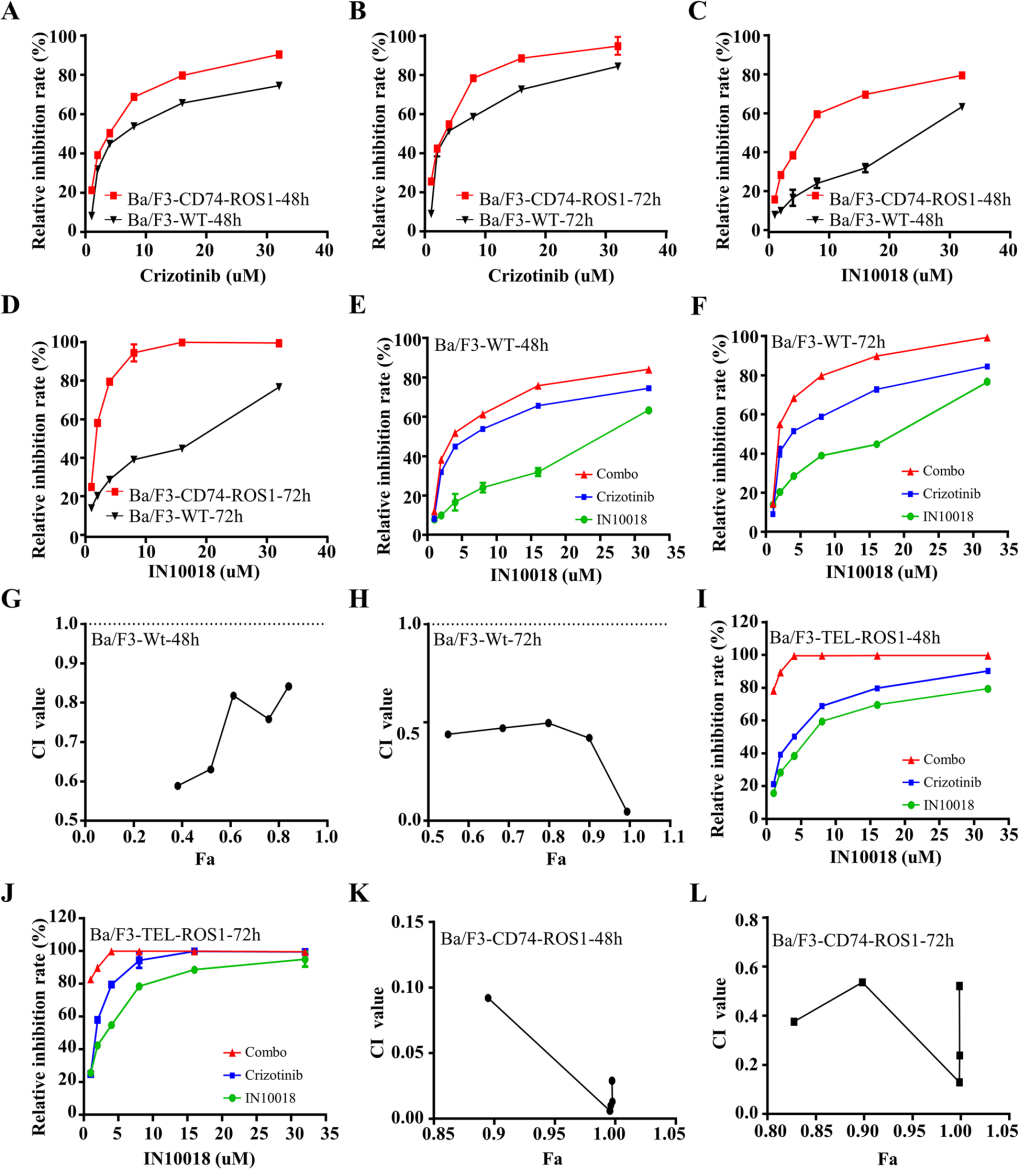
Crizotinib exhibited greater sensitivity towards Ba/F3-CD74-ROS1 cells compared to Ba/F3-WT cells, and its efficacy was enhanced when combined with IN10018. **A**, **B** Dose–response curves for the proliferation of Ba/F3-WT and Ba/F3-CD74-ROS1 cells treated with crizotinib for 48 h and 72 h, respectively. **C**, **D** Dose–response curves for the proliferation of Ba/F3-WT and Ba/F3-CD74-ROS1 cells treated with IN10018 for 48 h and 72 h, respectively. **E**, **F** The cell viability of IN10018 and crizotinib in Ba/F3-WT cells. The concentration ratio of IN10018 and Crizotinib is 2:1. **G**, **H** The CI value of Ba/F3-WT cells treated with IN10018, crizotinib, or combination therapy. **I**, **J** The cell viability of IN10018 and crizotinib in Ba/F3-CD74-ROS1 cells. The concentration ratio of IN10018 and Crizotinib is 2:1. **K**, **L** The CI value of Ba/F3-CD74-ROS1 cells treated with IN10018, crizotinib, or combination therapy. Significant levels: ns = not significant (*P* > 0.05), * *P* < 0.05, ** *P* < 0.01, and *** *P* < 0.001

Firstly, we assessed the proliferation of Ba/F3-WT cells and Ba/F3-CD74-ROS1 cells following treatment with crizotinib or IN10018, respectively. At both 48 and 72 h, Ba/F3-CD74-ROS1 cells exhibited greater sensitivity to crizotinib compared to Ba/F3-WT cells (Fig. 3A, B). Interestingly, Ba/F3-CD74-ROS1 cells also displayed higher sensitivity to IN10018 than Ba/F3-WT cells (Fig. 3C, D). These findings suggest that cells with elevated ROS1 expression are responsive to FAK inhibitors. Secondly, we compared the effects of combination therapy and individual inhibitors on Ba/F3-WT cells and Ba/F3-CD74-ROS1 cells. The combination therapy showed a stronger inhibitory effect on Ba/F3-WT cell proliferation compared to crizotinib or IN10018 alone (Fig. 3E, F). CI values below 1 indicated synergistic effects (Fig. 3G, H). Similar results were observed in Ba/F3-CD74-ROS1 cells (Fig. 3I-L), with a more pronounced inhibitory effect than in Ba/F3-WT cells. Our results illustrated the strong inhibitory impact of crizotinib on ROS1 and the synergistic effect when combined with IN10018.

### The combination of IN10018 and crizotinib suppressed proliferation and promoted apoptosis in TNBC cells

**Fig. 4:**
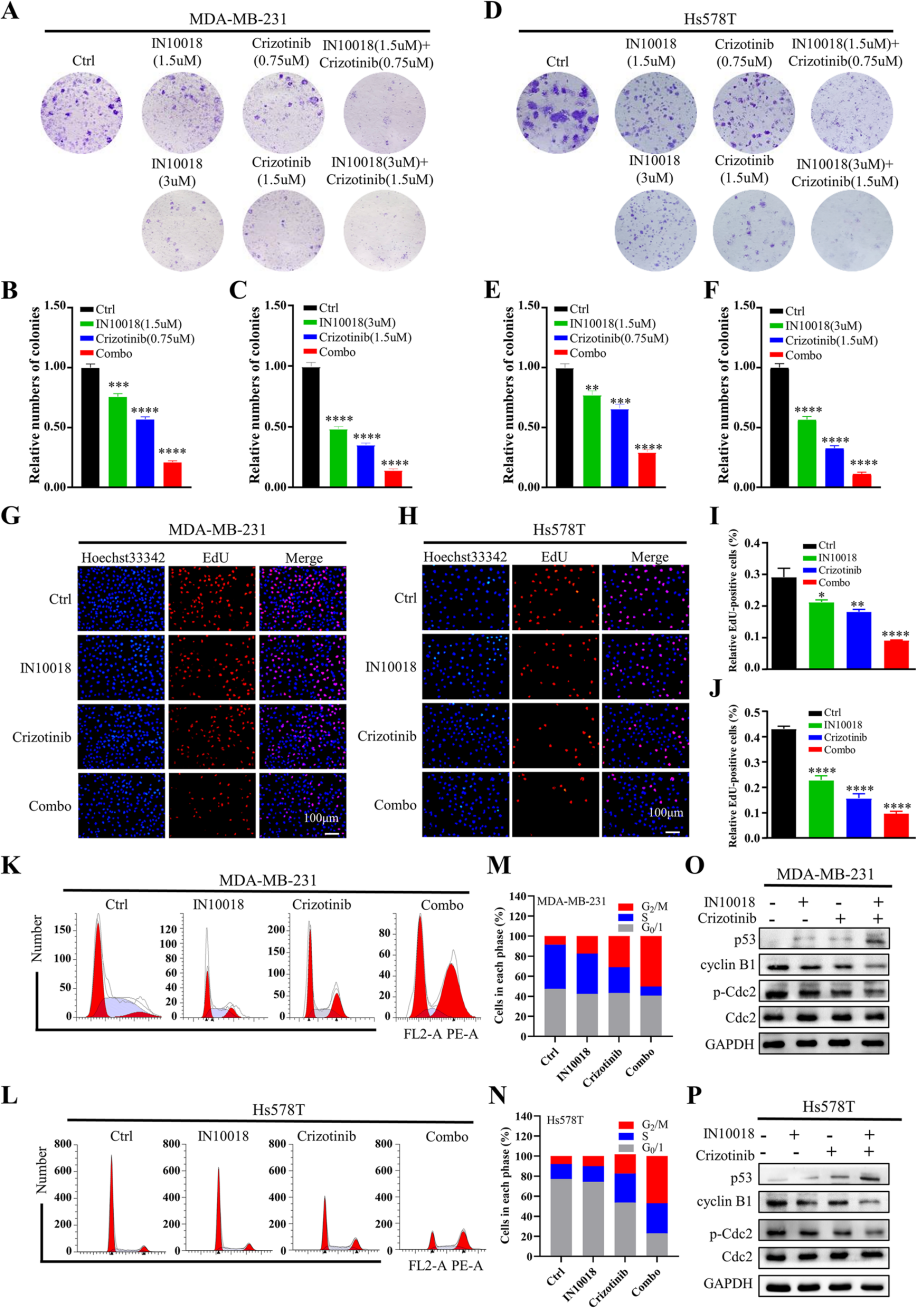
The combination of IN10018 and crizotinib effectively suppressed the proliferation of TNBC cells. **A** Colony formation and quantitative analysis of MDA-MB-231 cells. **B, C** Quantitative analysis of colony formation of MDA-MB-231 cells. **D** Colony formation of Hs578T cells. **E, F** Quantitative analysis of colony formation of Hs578T cells. **G**, **H** EdU staining results of MDA-MB-231 and Hs578T cells. **I**, **J** Quantitative analysis of EdU-positive cells. **K**, **L** Cell cycle analysis of MDA-MB-231 and Hs578T cells. **M, N** Quantitative analysis of cell cycle distribution. **O**, **P** Western blotting assessment of cell cycle-related proteins in MDA-MB-231 cells and Hs578T cells. Data are represented as mean ± SEM. Significant levels: ns = not significant (*P* > 0.05), * *P* < 0.05, ** *P* < 0.01, and *** *P* < 0.001. Ctrl: Control

To further demonstrate the synergistic anticancer effects of IN10018 and crizotinib, we conducted a series of in vitro experiments. Firstly, colony formation assays demonstrated that the combination of IN10018 and crizotinib exhibited superior inhibition of colony formation compared to crizotinib or IN10018 alone in MDA-MB-231 (Fig. 4A-C) and Hs578T (Fig. 4D-F) cells. Secondly, the EdU assay (Fig. 4G, H) demonstrated that the combination group significantly reduced the percentage of EdU-positive cells compared to the single-agent groups, indicating a substantial inhibition of cell proliferation (Fig. 4I, J). Thirdly, flow cytometry analysis (Fig. 4K, L) and quantitative analysis (Fig. 4M, N) of cell cycle distribution showed that combination therapy results in G_2_/M arrest in MDA-MB-231 and Hs578T cells. In addition, western blotting revealed an increase in p53 and a decrease in cyclin B1 and p-Cdc2 (Fig. 4O, P), which were crucial proteins involved in promoting the transition of cells from the G_2_ phase into mitosis. These findings suggest that the combined use of IN10018 and crizotinib can effectively cause G_2_/M arrest, resulting in reduced cell proliferation.

**Fig. 5:**
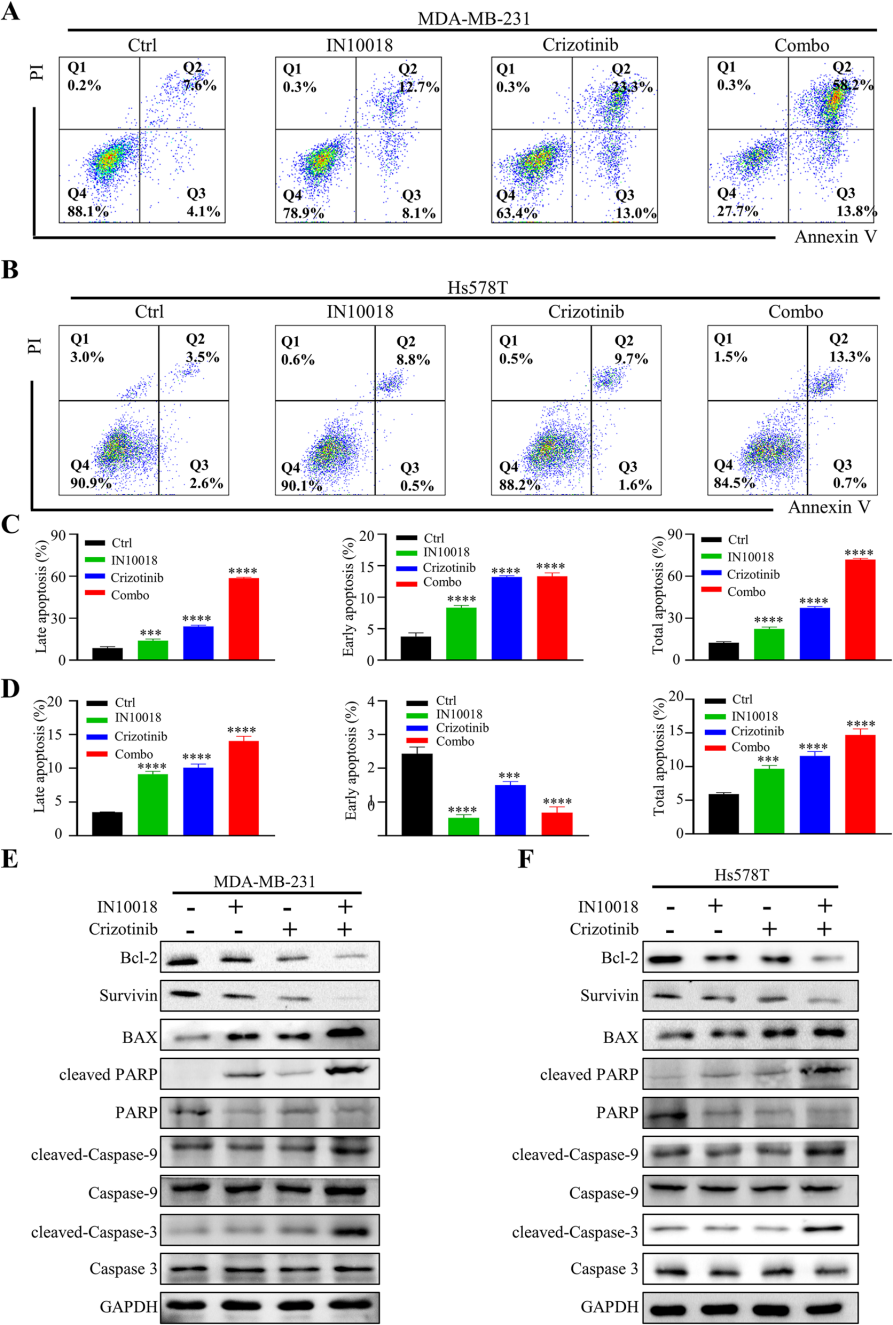
The combination of IN10018 and crizotinib promoted apoptosis in TNBC cells. **A**, **B** Representative flow cytometric plots of MDA-MB-231 and Hs578T cells. **C**, **D** Quantification of apoptosis rate of MDA-MB-231 and Hs578T cells. Q1: dead cells; Q2: late apoptosis; Q3: early apoptosis; Q4: living cells. **E**, **F** Western blotting assessment of cell apoptosis-related proteins in MDA-MB-231 and Hs578T cells. Data are represented as mean ± SEM. Significant levels: ns = not significant (*P* > 0.05), * *P* < 0.05, ** *P* < 0.01, and *** *P* < 0.001

In addition to their effects on the cell cycle, we aimed to explore the impact of drugs on apoptosis by flow cytometry analysis (Fig. 5A, B). Interestingly, when crizotinib or IN10018 were used individually, a slight increase in apoptosis was observed. However, when these two inhibitors were combined, a remarkable enhancement in total apoptosis was observed (Fig. 5C, D). To investigate the underlying mechanism of apoptosis induced by the combination of the combination therapy, western blotting was conducted to assess the levels of apoptosis-related proteins (Fig. 5E, F). The results revealed up-regulation of pro-apoptotic proteins, including cleaved-Caspase-3, cleaved-Caspase-9, cleaved PARP, and BAX, while down-regulation of anti-apoptotic proteins, such as Bcl-2 and survivin, was observed in the combination therapy.

### Combining IN10018 with crizotinib suppressed the growth of the TNBC xenograft models

**Fig. 6:**
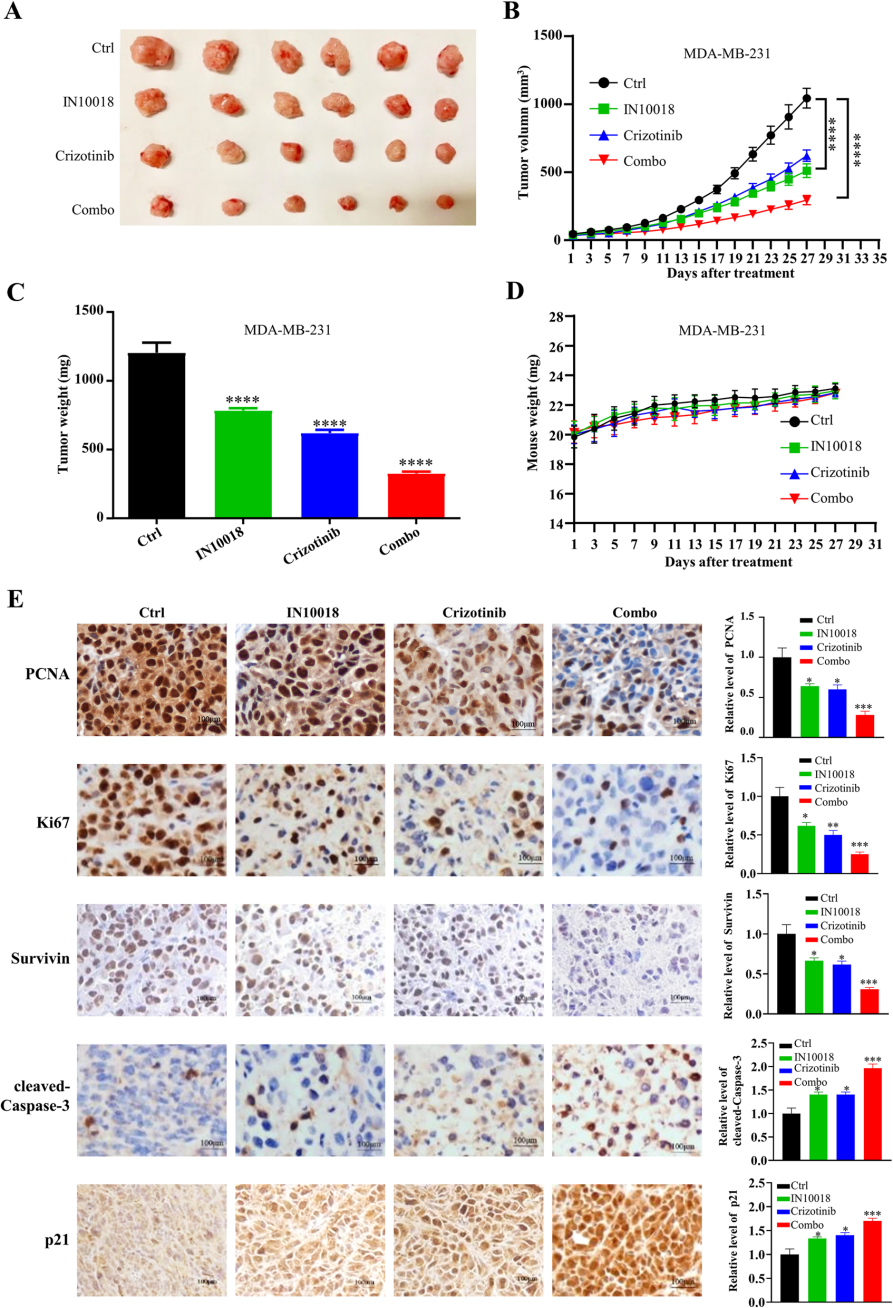
Combining IN10018 with crizotinib suppressed the growth of the TNBC xenograft models. The control group was treated with 0.5% natrosol 250 HX by intragastric administration once a day. IN10018 or crizotinib group were given intragastric administration of 25 mg/kg once a day. **A** Representative images of MDA-MB-231 tumors. **B** Growth curves of MDA-MB-231 tumors. **C** Tumor weights of MDA-MB-231 tumors. **D** Mouse weight of MDA-MB-231 tumors. **E** Immunohistochemical staining and quantitative analysis of the subcutaneous tumor tissues. Data are represented as mean ± SEM. Significant levels: ns = not significant (*P* > 0.05), * *P* < 0.05, ** *P* < 0.01, and *** *P* < 0.001

To evaluate the synergistic anticancer effect of combining crizotinib with IN10018 in vivo, we established MDA-MB-231 xenograft models. The mice were randomly divided into four groups: control, crizotinib, IN10018, and combination treatment. The control group received daily gavage administration of 0.5% naltrexone (250 HX). The crizotinib or IN10018 group received daily gavage administration with a dosage of 25 mg/kg. In TNBC xenograft models, the tumor volume in the combination therapy group was 73% smaller compared to the control group (Fig. 6A, B). Consistent with the tumor growth curves, the tumor weight reflected a similar trend (Fig. 6C). There were no differences in mouse weight between the groups (Fig. 6D). After euthanizing the mice, immunohistochemical analysis was performed on the tumor tissues (Fig. 6E). The proliferation markers Ki67 and PCNA, indicative of tumor proliferation, were significantly reduced in the combination treatment group, while the cell proliferation inhibitory protein p21 was substantially increased. Moreover, the level of the anti-apoptotic protein survivin was simultaneously decreased, while the level of the apoptosis-activating protein cleaved-Caspase-3 was increased in the combined group. These findings suggest the combination group effectively inhibits tumor growth, suppresses proliferation, and promotes apoptosis compared to the monotherapy group.

### Crizotinib combined with IN10018 exhibited a synergistic effect in human TNBC organoids

**Fig. 7:**
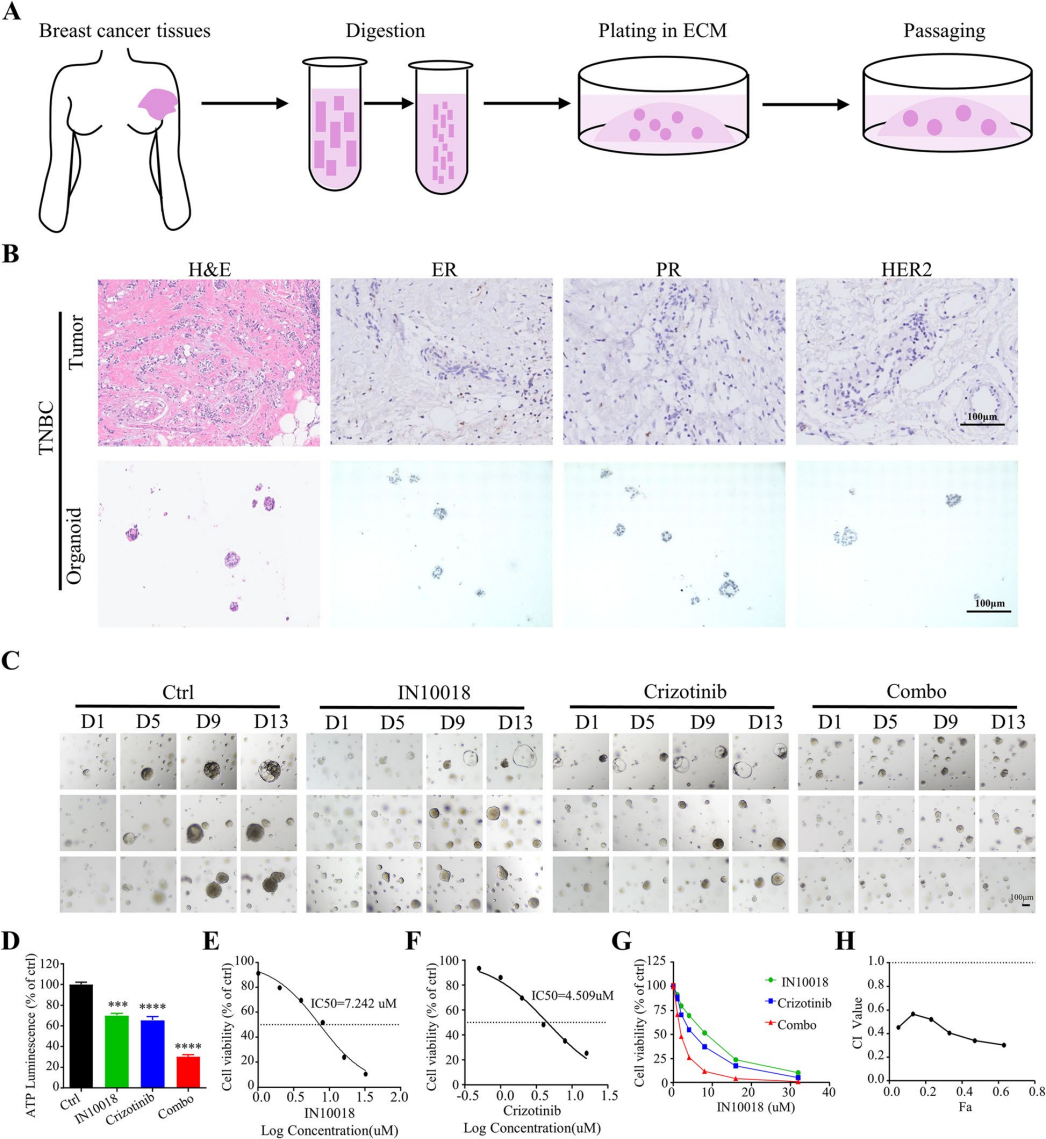
Crizotinib combined with IN10018 exhibited a synergistic effect in human TNBC organoids. **A** Steps for constructing TNBC organoids. **B** H&E staining and immunohistochemistry of TNBC tissues and organoids. **C** Bright-field images of TNBC organoids after being treated with IN10018, crizotinib, or combination therapy. Scale bar, 50 µm. **D** Quantification of organoid proliferation by CellTiter-Glo assay. **E**, **F** The cell viability and IC50 of TNBC organoids. **G** Survival curves of TNBC organoids. The concentration ratio of IN10018 and Crizotinib is 2:1. **H** The CI values of TNBC organoids. Data are represented as mean ± SEM. Significant levels: ns = not significant (*P* > 0.05), * *P* < 0.05, ** *P* < 0.01, and *** *P* < 0.001

We successfully established human TNBC organoids by collecting surgical specimens (Fig. 7A). To verify whether the TNBC organoids accurately represented the original histology, we performed H&E and histopathological analyses of the tissue and the organoids (Fig. 7B). The results demonstrated that the TNBC organoids exhibited morphological consistency with the original tumor. Furthermore, key breast cancer biomarkers, including ER, PR, and HER2, displayed consistent expression patterns in both the organoids and primary tumors. Subsequently, we evaluated the efficacy of IN10018 and crizotinib using TNBC organoids. The combination therapy resulted in a 70% reduction in cell viability in human TNBC organoid models (Fig. 7C-G). These findings are consistent with the results of in vivo and in vitro cellular experiments. To quantitatively assess the synergistic effect of the two drugs, we calculated the CI values (Fig. 7H), which were all below 1, indicating the synergistic effect between the two inhibitors. The results of human TNBC organoids provide compelling evidence supporting the clinical application of the synergistic inhibition achieved through the combination of IN10018 and crizotinib.

### Combination of crizotinib with IN10018 led to an enrichment of ROS, p53, and the GSH metabolic pathway

**Fig. 8:**
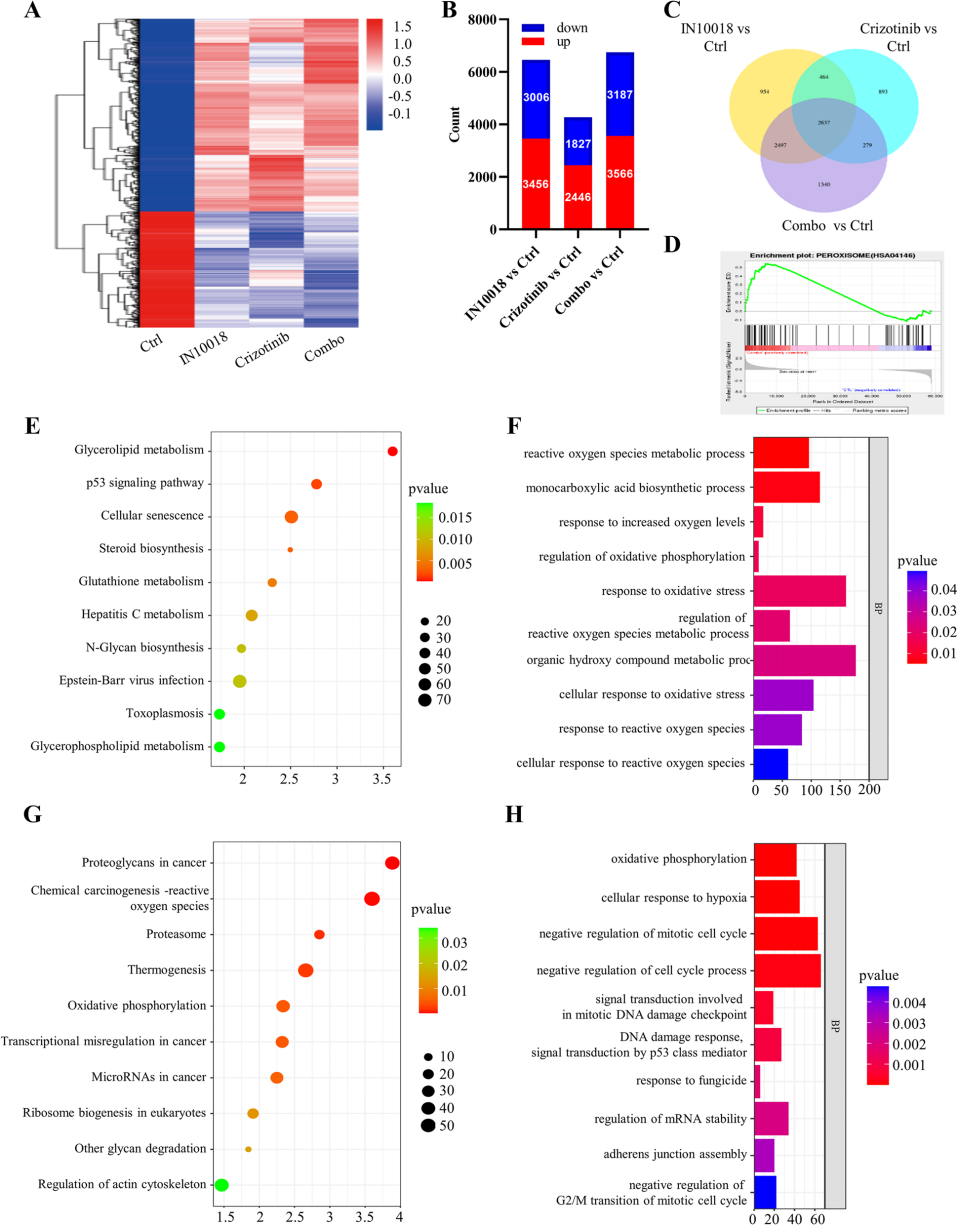
Combination of crizotinib with IN10018 led to an enrichment of ROS, p53, and the GSH metabolic pathway. **A** The heatmap of MDA-MB-231cells treated with IN10018 and ROS1 inhibitors by RNA-seq. **B** Histograms of the number of up-regulated (red bars) and down-regulated (blue bars) genes in different groups of MDA-MB-231 cells. **C** Venn diagram displaying the overlaps among different groups of MDA-MB-231 cells. **D** Genomic enrichment analysis (GSEA) identified significant changes in the combined group of MDA-MB-231 cells compared to the control group. **E** KEGG analysis results were shown by a bubble diagram of MDA-MB-231 cells. **F** GO analysis results were shown by a bar diagram of MDA-MB-231 cells. **G** KEGG analysis results were shown by a bubble diagram of MDA-MB-231 tumors. **H** GO analysis results were shown by a bar diagram of MDA-MB-231 cells. Significant levels: ns = not significant (*P* > 0.05), * *P* < 0.05, ** *P* < 0.01, and *** *P* < 0.001

MDA-MB-231 cells were treated with IC50 concentrations of IN10018 and crizotinib for 24 h. RNA-seq analysis of TNBC cells revealed significant gene expression changes in the experimental group compared to the untreated group (Fig. 8A-C). The combination group exhibited enrichment of the peroxisome pathway, as indicated by GSEA analysis (Fig. 8D). This suggests that the combination therapy affects cellular responses to oxidative stress, as peroxisomes are involved in regulating ROS and metabolizing oxidative stress products. The analysis of the KEGG pathway in MDA-MB-231 cells, treated with IN10018 and crizotinib, unveiled a significant influence of the combination on both the p53 pathway and GSH metabolism (Fig. 8E). Additionally, GO analysis indicated that the combination therapy influenced the cellular response to ROS, indicating its ability to modulate the cell's response to oxidative stress and enhance its anti-tumor effects (Fig. 8F). Additionally, RNA-seq analysis of MDA-MB-231 xenograft tumor tissues showed alterations in the cellular response to ROS due to the combination of the two drugs, as indicated by KEGG pathway analysis (Fig. 8G). Furthermore, the GO analysis demonstrated significant enrichment of oxidative phosphorylation in the combined group (Fig. 8H).

### Ferroptosis induced by combining crizotinib with IN10018 was associated with p53

**Fig. 9:**
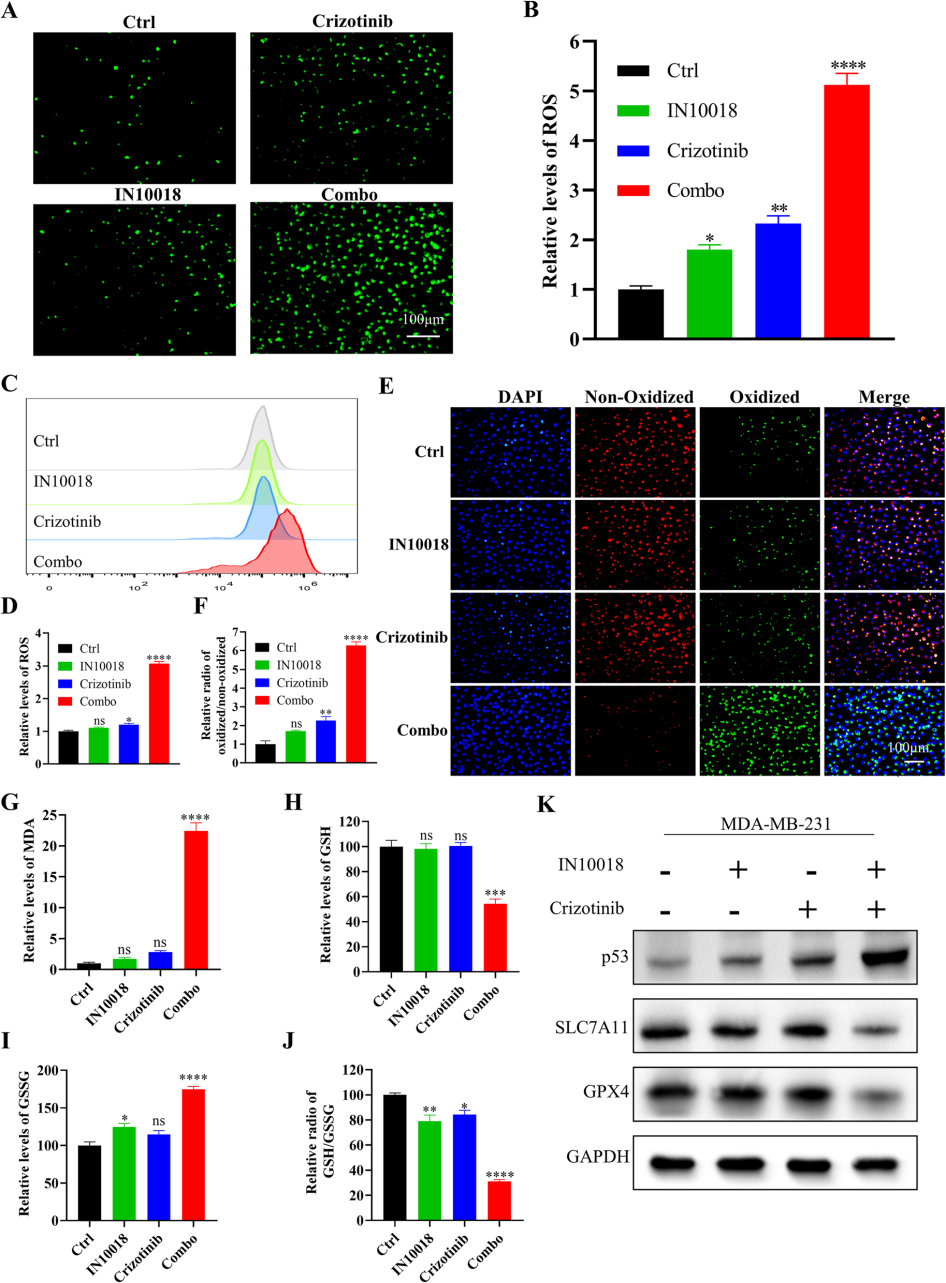
Combined IN10018 and crizotinib induced ferroptosis. **A** Fluorescence pictures of ROS. **B** Quantitative analysis of ROS fluorescence intensity. **C** ROS detection by Flow cytometry. **D** Quantitative analysis of ROS. **E** Measurement of lipid peroxidation using C11-BODIPY. **F** Quantitative analysis of lipid peroxidation using C11-BODIPY. **G** MDA content assay. **H**, **I**, **J** GSH and GSSG concentrations were determined using a GSH assay kit. **K** Western blotting results for ferroptosis-related proteins. Significant levels: ns = not significant (*P* > 0.05), * *P* < 0.05, ** *P* < 0.01, and *** *P* < 0.001

**Fig. 10:**
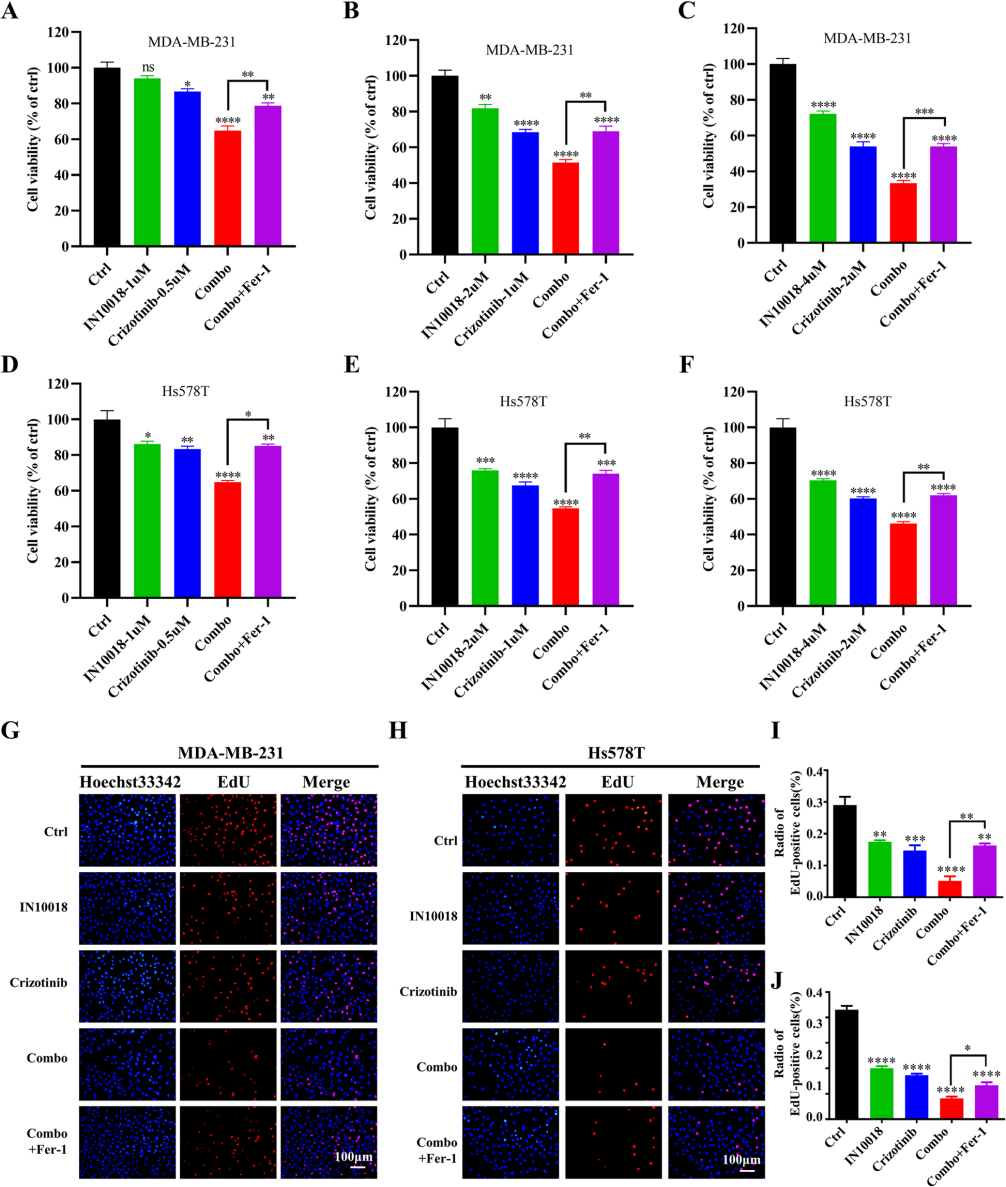
Fer-1 restored the proliferative capacity of combined group cells. **A**-**C** The cell viability of MDA-MB-231 cells treated with IN10018, crizotinib, and 1 uM of Fer-1 for 24 h. **D**-**F** The cell viability of Hs578T cells treated with IN10018, crizotinib, and 1 uM of Fer-1 for 24 h. **G**, **H** EdU staining results of MDA-MB-231 and Hs578T cells. **I, J** Quantitative analysis of EdU-positive cells. Significant levels: ns = not significant (*P* > 0.05), * *P* < 0.05, ** *P* < 0.01, and *** *P* < 0.001

RNAseq results suggested synergistic effect of the combination therapy was associated with ferroptosis, a newly identified type of cell death characterized by lipid peroxidation and GSH production. MDA-MB-231 cells were treated with IC50 concentrations of IN10018 and crizotinib for 24 h. Immunofluorescence staining for ROS revealed a fivefold rise in ROS levels in the combination therapy compared to IN10018 and crizotinib alone (Fig. 9A, B). Flow cytometry analysis of ROS levels also revealed higher levels of apoptosis in the combination therapy (Fig. 9C, D). In addition, a sixfold increase in lipid peroxidation were detected in combined group. While IN10018 alone resulted in slight changes, the combination therapy led to a significant increase in lipid peroxidation (Fig. 9E, F). The lipid peroxidation product, MDA, was also significantly elevated in the combined group (Fig. 9G). We used a GSH assay kit to further explore the effect of combination therapy on GSH levels. There was no significant effect on GSH and GSSG levels when IN10018 or crizotinib were administered alone. However, there was a significant reduction in GSH levels in the combination group (Fig. 9H), an increase in GSSG (Fig. 9I), and a 69% decrease in the GSH/GSSG ratio (Fig. 9J). Western blotting results demonstrated a significant decrease in the expression of SLC7A11 and GPX4 proteins in the combination therapy group, along with the upregulation of the p53 expression (Fig. 9K). To further demonstrate the efficacy of the combination therapy in inducing ferroptosis, we treated the cells with IN10018, crizotinib, and 1 μM of Fer-1 for 24 h. The cell counting results of MDA231 (Fig. 10A-C) and Hs578T (Fig. 10D-F) demonstrated that the addition of Fer-1 reversed cell proliferation. Similar results were also observed in EdU assays (Fig. 10G-J).

**Fig. 11:**
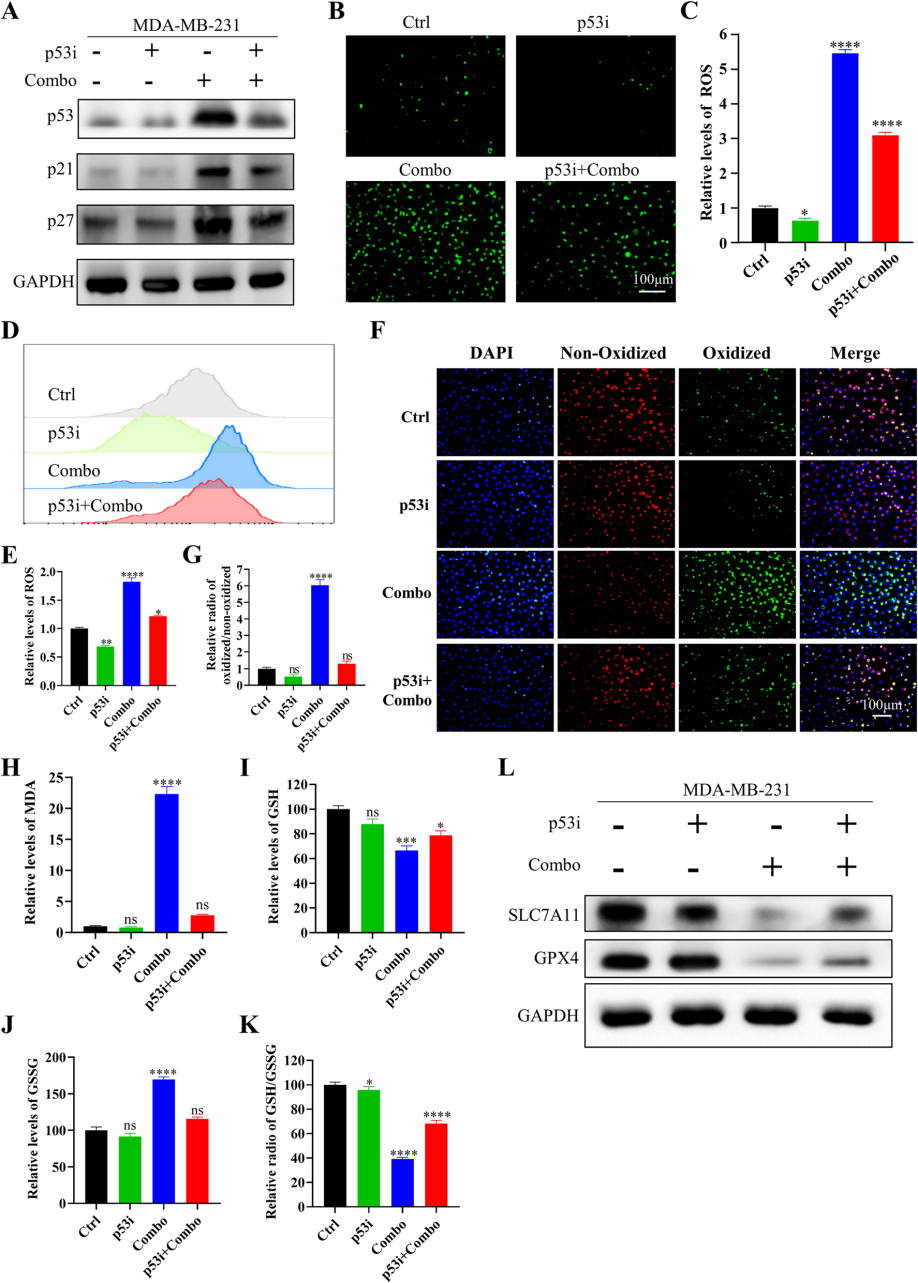
Ferroptosis induced by combining crizotinib with IN10018 was associated with p53. **A** Western blotting results of p53 downstream target proteins. **B** Fluorescence pictures of ROS. **C** Quantitative analysis of ROS fluorescence intensity. **D** ROS detection by Flow cytometry. **E** Quantitative analysis of ROS. **F** Measurement of lipid peroxidation using C11-BODIPY. **G** Quantitative analysis of lipid peroxidation using C11-BODIPY. **H** MDA content assay. **I**, **J**, **K** GSH and GSSG concentrations were determined using a GSH assay kit. **L** Western blotting results for ferroptosis-related proteins. Significant levels: ns = not significant (*P* > 0.05), * *P* < 0.05, ** *P* < 0.01, and *** *P* < 0.001

We aim to investigate the role of p53 in the induction of ferroptosis by combination therapy. Firstly, the cells were subjected to a 24-h treatment with a p53 inhibitor, Pifithrin-α, at a concentration of 10uM. Western blotting analysis demonstrated a down-regulation of p53, as well as a decrease in the expression levels of its downstream genes, p21, and p27 (Fig. 11A). Moreover, immunofluorescence staining and flow cytometry analysis of ROS levels demonstrated that the addition of a p53 inhibitor in the combination therapy group result in a notable decline in ROS levels compared to the combination therapy group (Fig. 11B-E). Additionally, the lipid peroxidation (Fig. 11F, G) and MDA assay (Fig. 11H) results showed a notable reduction in lipid peroxidized cells and MDA content upon the addition of the p53 inhibitor in the combination treatment group, as compared to the combination group. Moreover, when added to the combination therapy group, the p53 inhibitor increased the GSH/GSSG ratio (Fig. 11I-K). Western blotting assay results (Fig. 11L) demonstrated that the addition of the Pifithrin-α in the combination group caused a decline in the expression of SLC7A11 and GPX4, which are upregulated by the combination therapy.

## Discussion

TNBC presents a significant challenge due to its heterogeneity and limited targeted therapy options [8]. A novel approach to targeted therapy involves exploiting synthetic lethality, exemplified by the use of PARP inhibitors in TNBC patients with BRCA1 deficiency [39, 40]. Recent research has uncovered the potential of inhibiting ROS1 with crizotinib to induce synthetic lethality in TNBC patients with E-cadherin deficiency [13], though resistance to crizotinib often hampers treatment effectiveness. Our study unveiled elevated FAK and ROS1 levels in breast cancer tissue compared to normal breast tissue. Notably, patients with increased expression of both FAK and ROS1 had the poorest prognosis, further emphasizing their role in TNBC. Additionally, we observed an upregulation of p-FAK in crizotinib-resistant TNBC. These findings strongly support the simultaneous inhibition of FAK and ROS1 in TNBC. In vitro experiments demonstrated a synergistic effect in inhibiting TNBC cell proliferation and promoting apoptosis when combining IN10018 and crizotinib. Furthermore, we observed synergistic anti-tumor effects of these inhibitors in TNBC xenograft models and human TNBC organoid models. RNA-seq analysis indicated that DEGs were associated with oxidative stress responses and influenced GSH metabolism and p53 signaling pathways. The combination therapy elevated ROS, lipid peroxides, and MDA, known inducers of ferroptosis while reducing GSH levels, inhibiting ferroptosis. Western blotting analysis revealed decreased SLC7A11 and GPX4 levels, along with increased p53 expression in the combination group. Importantly, the use of a p53 inhibitor effectively reversed the ferroptosis phenotype induced by the combination therapy.

Targeting the cell cycle and apoptosis is crucial for inhibiting tumor proliferation. In vitro experiments highlighted that the combination of IN10018 and crizotinib synergistically inhibited proliferation and promoted apoptosis by upregulating p53 expression. This was associated with G_2_/M phase arrest and a reduction in the levels of cyclin B1 and p-Cdc2, which promote mitotic initiation. Additionally, we observed an upregulation of the pro-apoptotic gene BAX, a downregulation of the anti-apoptotic gene Bcl-2, and elevated the expression of cleaved-Caspase-3, cleaved-Caspase-9, and cleaved PARP. P53’s role in reducing cyclin B1 and p-Cdc2 levels, leading to G_2_/M arrest, has been previously documented [41]. Furthermore, p53 is a critical regulator of apoptosis [42], activating the mitochondria-mediated apoptotic pathway by increasing pro-apoptotic genes like BAX while inhibiting anti-apoptotic genes like Bcl-2 [43]. The upregulation of BAX triggers the activation of apoptotic proteins, such as cleaved-Caspase-9, subsequently stimulating cleaved-Caspase-3, PARP fragmentation, and the induction of apoptosis [44]. In summary, our combination treatment mechanistically upregulates p53, leading to G_2_/M cell cycle arrest and enhanced apoptosis by modulating the BAX/Bcl-2 ratio, providing a promising approach for TNBC therapy.

Ferroptosis, a novel form of cell death characterized by GSH depletion and lipid peroxidation [45–47], has gained attention due to its close association with various cancers [48]. Our RNA-seq analysis revealed that the combination therapy enriched pathways related to oxidative stress, GSH metabolism, and p53. Additionally, this treatment increased ROS levels, enhanced lipid peroxidation, and reduced the GSH/GSSG ratio. Western blotting analysis demonstrated the upregulation of p53 and the downregulation of SLC7A11 and GPX4 in the combination group. Importantly, the addition of a p53 inhibitor reversed these effects. Recent investigations have emphasized p53’s role in ferroptosis by repressing the transcription of SLC7A11 [49], leading to GSH depletion [50, 51]. GSH is a precursor for GPX4 synthesis [52], a critical enzyme involved in lipid peroxide elimination. Therefore, decreased GSH levels result in GPX4 depletion, leading to lipid peroxide accumulation and the initiation of ferroptosis. The promotion of ferroptosis by combination therapy can be explained through the inhibition of the SLC7A11/GSH/GPX4 pathway by upregulating p53.

Recent research has uncovered a connection between FAK and ferroptosis. In NSCLC cells, FOCAD inhibited FAK activity, enhancing sensitivity to ferroptosis [53]. Additionally, inhibition of FAK activity in ovarian [54, 55] and cervical cancers [56] led to decreased GSH levels. FAK also serves as a scaffold for the binding of p53 and Mdm2, regulating ferroptosis via p53 [57]. In breast cancer, immunohistochemical analysis revealed a positive correlation between FAK, p53 expression (*P* = 0.0002), and p53 mutations (*P* < 0.0001) [58]. Treatment of NSCLC with the FAK inhibitor PF-573228 increased p53 expression [59]. Similarly, FAK inhibition in gastric cancer cells enhanced p53’s transcriptional activity [60]. In head and neck squamous cell carcinoma cells, p-FAK levels increased only in cisplatin-resistant p53 mutant cells, while no such increase occurred in the wild-type cell line [61]. Our findings align with previous studies, demonstrating that treatment with IN10018 alone resulted in an upregulation of p53 expression and downregulation of GSH levels, with more pronounced effects in the combined treatment group.

Emerging research highlights the pivotal role of p53 in predicting patient outcomes after treatment with ROS1 inhibitors [62]. In ROS1-positive NSCLC patients, treatment with the ROS1 inhibitor crizotinib led to progression-free survival (PFS) of around 8.5 months for individuals with p53 gene mutations, whereas those with a wild-type p53 gene had a longer PFS of approximately 15.5 months [63]. A study on lorlatinib in ROS1-positive NSCLC patients showed that those with p53 mutations had a PFS of 8.5 months, while those with p53 wild-type had a PFS of approximately 23.6 months [64]. These findings suggest that p53 gene mutations may hinder the responsiveness of cancer cells to ROS1-targeted therapies. A recent study has demonstrated the potential of FAK inhibitors in disrupting the FAK-YAP-TRX signaling pathway, augmenting the effectiveness of ROS1 inhibitors in cancers [65]. Our research emphasizes the synergistic effects of co-administering IN10018 and crizotinib, which not only inhibit cell proliferation but also promote apoptosis and ferroptosis. These beneficial outcomes are directly associated with the upregulation of p53 expression, revealing a novel mechanism that distinguishes it from previously known modalities. Our experiments are consistent with clinical data, providing a scientific basis for the synergistic anti-tumor effect.

Accurate prediction of oncology drug sensitivity is crucial for guiding clinical drug utilization. Patient-derived cell lines or xenografts are commonly used for predicting drug efficacy but often fail in clinical trials [66, 67]. Long-term in vitro culture of cell lines leads to loss of original tumor characteristics and inaccurate replication of the tumor microenvironment [68]. Patient-derived xenograft (PDX) models maintain tumor heterogeneity but face challenges such as low transplantation rates, large differences in human and mouse drug-use behavior, and a long process of drug screening [69, 70]. The emergence of patient-derived tumor organoids (PDOs) addresses these challenges [71]. PDOs are 3D structures generated from tissue stem cells, accurately capturing tumor heterogeneity and restoring the tumor microenvironment [72]. In addition, PDOs have the advantage of being generated from a small number of biopsy samples, making them valuable for studying rare or difficult-to-obtain tumor types [73]. Importantly, PDOs accurately predict a patient's response to drugs and have become an essential preclinical model for assessing the efficacy of oncology drugs and enabling personalized cancer therapy [74, 75]. To further investigate the efficacy of IN10018 and crizotinib, we successfully established human TNBC organoids using established research techniques [76]. Our findings indicate that organoids resembling TNBC histological characteristics similar to the original tumors. Treatment with either IN10018 or crizotinib alone inhibited TNBC organoid growth in a dose-dependent manner. Importantly, the combination of IN10018 and crizotinib exhibited a synergistic effect in suppressing TNBC organoid proliferation. These experimental outcomes align with in vivo and in vitro studies, providing strong scientific support for the potential clinical application of this drug combination in treating TNBC.

**Fig. 12:**
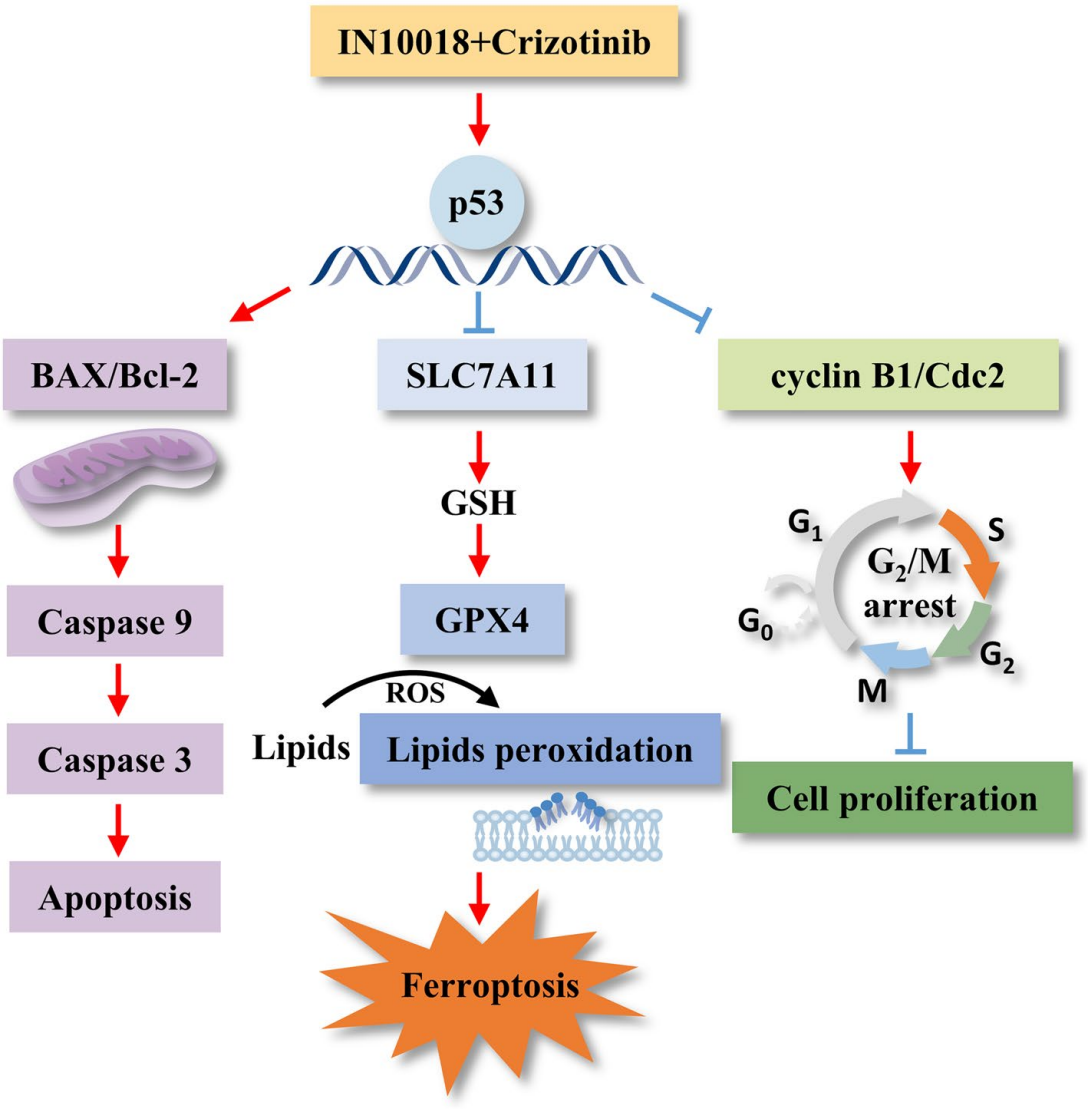
Schematic representation of the mechanism of the synergistic effect of crizotinib in combination with IN10018 in TNBC

In this study, we have effectively demonstrated the synergistic impact of combining IN10018 and crizotinib in TNBC. Mechanistically, the combination upregulated p53 inhibited cell proliferation by G_2_/M arrest, enhanced apoptosis by elevating the BAX/Bcl-2 ratio, and induced ferroptosis through the SLC7A11/GSH/GPX4 pathway (Fig. 12). Our study suggests a new approach to the treatment of TNBC. However, before implementing this innovative approach in clinical practice, a comprehensive pre-clinic study is required to evaluate the safety and effect of this combination.

## Data Availability

Not applicable.

## Abbreviations

CI: Combination index
Ctrl: Control
DCFH-DA: 2′,7′-Dichlorofluorescein diacetate
DEGs: Differentially expressed genes
EdU: 5-Ethynyl-2′-deoxyuridine assay
ECM: Extracellular matrix
FAK: Focal adhesion kinase
Fer-1: Ferrostatin-1
GEO: Gene Expression Omnibus
GO: Gene Ontology
GSH: Glutathione
GSSG: Oxidized glutathione
HER2: Human epidermal growth factor receptor 2
H: High
KEGG: Kyoto Encyclopedia of Genes and Genomes
L: Low
MDA: Malondialdehyde
NSCLC: Non-small cell lung cancer
PBS: Phosphate-buffered solution
PDOs: Patient-derived tumor organoids
PDX: Patient-derived xenograft
PFS: Progression-free survival
ROS1: ROS proto-oncogene 1
ROS: Reactive oxygen species
TNBC: Triple-negative breast cancer
T-GSH: Total glutathione
